# Neural Correlates of Telicity in Spanish-Speaking Children with and without Developmental Language Disorder

**DOI:** 10.3390/children11080982

**Published:** 2024-08-14

**Authors:** Mabel Urrutia, Soraya Sanhueza, Hipólito Marrero, Esteban J. Pino, María Troncoso-Seguel

**Affiliations:** 1 Facultad de Educación, Universidad de Concepción, Concepcion 4070386, Chile; 2 Facultad de Humanidades y Arte, Universidad de Concepción, Concepcion 4070386, Chile; sorayasanhueza@udec.cl; 3 Facultad de Psicología y Logopedia, Instituto Universitario de Neurociencia de la Universidad de La Laguna (IUNE), Universidad de La Laguna, 38200 Santa Cruz de Tenerife, Spain; hmarrero@ull.es; 4 Facultad de Ingeniería, Universidad de Concepción, Concepcion 4070386, Chile; estebanpino@udec.cl (E.J.P.); maria.troncoso@biomedica.udec.cl (M.T.-S.)

**Keywords:** telicity, Developmental Language Disorder, verb recognition, Event-Related Potential, N400, P600, lexical aspect, semantic incongruity

## Abstract

Background: It is broadly acknowledged that children with Developmental Language Disorder (DLD) show verb-related limitations. While most previous studies have focused on tense, the mastery of lexical aspect—particularly telicity—has not been the primary focus of much research. Lexical aspect refers to whether an action has a defined endpoint (telic verbs) or not (atelic verbs). Objective: This study investigates the effect of telicity on verb recognition in Chilean children with DLD compared to their typically developing (TD) peers using the Event-Related Potential (ERP) technique. Method: The research design is a mixed factorial design with between-group factors of 2 (DLD/TD) and within-group factors of 2 (telic/atelic verbs) and 2 (coherent/incoherent sentences). The participants were 36 school-aged children (18 DLD, 18 TD) aged 7 to 7 years and 11 months. The task required subjects to listen to sentences that either matched or did not match an action in a video, with sentences including telic or atelic verbs. Results: The study found notable differences between groups in how they processed verbs (N400 and post-N400 components) and direct objects (N400 and P600 components). Conclusions: Children with DLD struggled to differentiate telic and atelic verbs, potentially because they employed overgeneralization strategies consistent with the Event Structural Bootstrapping model.

## 1. Introduction

Language acquisition is a complex and continuous aspect of human development that begins in early childhood and progresses through various milestones over time. Developmental Language Disorder (DLD) is a neurodevelopmental disorder that disrupts this process, resulting in persistent difficulties in acquiring and using language at different linguistic levels, including phonological, morphological, syntax, lexical, and semantic. In this research, we will focus on the lexical–semantic levels, which pose a major challenge for this type of disorder [1].

The diagnosis of DLD is independent of sensory, neurological, or intellectual impairments [1]. DLD typically emerges around the age of three and can significantly affect both language comprehension and production, resulting in ongoing difficulties in social integration and academic performance, regardless of the language spoken [2,3,4]. DLD is a globally recognized concern within special education, where the importance of early identification and the implementation of specialized interventions is widely recognized [1,5].

The neural bases of DLD involve several findings related to brain structure and function. Children with DLD exhibit differences in overall brain volume, with some studies reporting smaller or larger volumes compared to neurotypical children [6,7]. Alterations in both gray and white matter volume are also observed [8], including increased white matter volumes in younger children, potentially indicating inefficient neural connectivity. Specific regions, such as the planum temporale, which is associated with language functions and is typically larger in the left hemisphere in neurotypical individuals, do not show consistent asymmetry in children with DLD [9]. The inferior frontal gyrus (IFG), crucial for language production, also exhibits abnormal activation and volume patterns [10,11]. The caudate nucleus, a subcortical structure involved in language processing and verbal memory, shows reduced gray matter volume. White matter tract integrity, particularly in the superior longitudinal fasciculus and arcuate fasciculus, is also compromised, affecting language processing and production. Functional MRI studies reveal atypical brain activation patterns, including hypoactivation in the left IFG and compensatory activation in the right IFG [9]. Additionally, children with DLD exhibit reduced cerebral blood flow in the left Perisylvian regions, negatively impacting language processing [12]. These findings indicate that DLD is associated with structural and functional alterations in key language-related brain regions and their connections, contributing to the linguistic difficulties observed in affected children.

Among the linguistic levels affected by DLD, the semantic aspect, including the acquisition and use of verbs, is particularly crucial. Verbs are not only semantically significant, but also serve as the backbone of grammatical structure, making their mastery essential for constructing sentences and conveying actions effectively [13]. The ability to acquire, comprehend, and accurately use verbs is central to language development because it underpins the formation of complex sentences and overall syntactic organization. Research has demonstrated that a robust verb vocabulary is a key predictor of linguistic outcomes, surpassing the influence of noun knowledge in both typically developing children and those with DLD [14]. Moreover, errors in verb usage have been consistently identified as clinical markers of DLD across various languages, including English, German, and Spanish [15,16,17,18].

One aspect of verb usage that has garnered recent research attention is telicity, which refers to the inherent endpoint of an action. Telicity is a subcategory of aspect, a grammatical category that deals with the temporal structure of events. Aspect is divided into lexical aspect and grammatical aspect. Lexical aspect, which includes telic and atelic distinctions, refers to the inherent temporal properties of the verb itself. Telic verbs denote actions with a clear endpoint (e.g., “close”, “break”), while atelic verbs describe actions without a defined conclusion (e.g., “run”, “paint”). Grammatical aspect, on the other hand, involves the use of morphological markers that indicate whether an action is completed (perfective) or ongoing (imperfective) [19]. For the purposes of this investigation, we will study the lexical aspect associated with types of verbs, such as telic and atelic.

Studies conducted with English- and German-speaking populations have shown that children with DLD struggle to associate telic verbs with completed actions, a skill that typically developing children acquire early on. In Spanish, as in these other languages, telicity is primarily encoded lexically, meaning that it is embedded within the verb itself rather than systematically marked by grammatical structures [17,20,21]. This difficulty with processing the lexical aspect of verbs suggests that the challenges in verb usage for children with DLD are deeply rooted in the inherent semantic properties of verbs, making it a critical area for further research [22].

Understanding how children learn to process telic and atelic verbs can be further explained through the Event Structural Bootstrapping model [22]. This model posits that children focus on event structure when learning verbs, initially acquiring those with clear final states (telic verbs) to facilitate semantic integration. In contrast, atelic verbs, with their ambiguous endpoints, present greater learning challenges and are acquired later. Studies comparing children with DLD and TD children speaking English and German support this model, showing that TD children process telic verbs more efficiently than atelic verbs, a difference not observed in DLD populations [20,22,23]. Schulz [17] suggests that this deficit might be universal, and Leonard [19] hypothesizes that the difficulty in identifying the telicity of verbs may underlie future verb conjugation errors in individuals with DLD.

Currently, research on telicity in Spanish-speaking children with DLD is limited, as we found only one significant study on this topic related to speech production. In this regard, Grinstead et al. [24] analyzed the spontaneous speech of 38 Mexican children (19 with DLD and 19 typically developing) with a mean age of 6 years. Their findings revealed a general preference in both groups for the use of telic verbs in the past tense and atelic verbs in the present tense, a pattern consistent with what has been observed in other languages [25,26]. While children with DLD produced fewer verbs overall than their typically developing peers, no significant differences were found in the use of lexical aspect between the groups.

In the area of language comprehension, we found one study on a DLD population of Spanish speakers by Christou et al. [27]. The manipulation in this study consisted of present- and future-tense sentences with specific Spanish morphological markers. Similar to Grinstead’s study, the authors found no significant differences between the DLD population and typically developing children using the eye-tracking technique. These findings highlight the need to use more direct techniques to determine language processing in the population under study.

This study aims to address this gap by examining the effect of telicity in Spanish-speaking children with DLD compared to their TD peers using the Event-Related Potential (ERP) technique. Verbs play a central role in language, serving as the backbone of sentence structure and meaning. ERP provides a direct and time-sensitive measure of brain activity, allowing us to explore the neural mechanisms underlying language processing, particularly how these crucial elements of language are handled by the brain [28]. Specifically, by analyzing the N400 component—a negative-going wave that peaks around 400 milliseconds after stimulus onset and is associated with the processing of meaning—we can gain deeper insights into how children with DLD process telic and atelic verbs.

Previous studies [29] have shown that TD children exhibit a strong N400 effect in earlier time windows (300–500 ms and 500–800 ms), while children with DLD only show a reliable N400 effect in the later window (500–800 ms). Additionally, the topographical distribution of N400 in children with DLD is less focalized compared to TD children, who show typical posterior effects. Courteau et al. [30] found similar centroparietal N400 effects in preadolescent French children with DLD, although with a brief delay in onset compared to their TD peers. Although the findings are different, we expect that the differences in the N400 component between telic and atelic verbs will provide a deeper understanding of how children with DLD process these linguistic features compared to their TD peers.

Our hypothesis is that school-aged children with DLD will show a delayed N400 effect in response to incoherent sentences, with no difference between telic and atelic verbs. In contrast, typically developing school-aged children will exhibit a larger N400 component for incoherent sentences with atelic verbs compared to those with telic verbs, as atelic verbs are processed differently.

## 2. Materials and Methods

The research design is experimental, using a mixed factorial approach. The design included one between-group factor (subjects with DLD/TD subjects) and two within-group factors (telicity: telic/atelic; and coherence: coherent/incoherent).

### 2.1. Participants

The study included 38 school-aged children, 19 with DLD (10 boys, 9 girls) and 19 TD (9 boys, 10 girls), who were finishing the second year of primary school. Initially, 38 subjects were recruited; however, one subject from each group was excluded during the analysis phase due to excessive artifacts in their EEG recordings, resulting in 18 participants per group (Table 1). For the estimation of the minimum required sample size, the following parameters were considered: (a) effect size (f) = 0.25; (b) statistical power (1 − β) = 0.95; (c) significance level (α) = 0.05; (d) number of measurements = 4. According to these variables, a minimum of 18 individuals per group was needed, as calculated by the G*Power program version 3.1.7. [31].

The children were recruited from subsidized private schools in Concepción (Chile), and all were native Spanish speakers. Written consent was obtained from their parents and/or legal guardians, and the participants provided verbal assent prior to participating in the experiment. This study was approved by the Ethics, Bioethics, and Biosafety Committee (Protocol No. CEBB 731-2020) at the University of Concepción (Chile).

Participants were selected based on specific inclusion criteria, ensuring that all children were attending school, were right-handed, and had normal or corrected vision. The diagnosis of DLD was made using the Chilean IDTEL instrument [32], which assesses language in children between the ages of 6 years and 9 years and 11 months, and was developed and validated in Chile. The test uses oral response items to assess the 4 levels of language: phonological, morphosyntactic, semantic, and pragmatic. It was administered by a single speech-language pathologist. Subjects in the DLD group had to score below the cut-off for the full test, and TD subjects had to score above the cut-off. Details of the sample are shown in Table 1.

Participants were excluded if they had a known sensory or developmental disorder, such as autism, cognitive impairment, cerebral palsy, or attention-deficit disorder. This information was verified through interviews with parents or caregivers.

### 2.2. Stimuli and Procedure

#### 2.2.1. Linguistic Material

Thirty-two telic verbs and thirty-two atelic verbs were used, which in counterbalance allowed for the creation of sixty-four sentences for each subject. These sentences followed the subject–verb–object structure and were divided into four categories: sixteen telic–coherent, sixteen telic–incoherent, sixteen atelic–coherent, and sixteen atelic–incoherent.

For each sentence, a video script was created to ensure that the video was either coherent or incoherent with the sentence. Care was taken to include the same characters and objects within each variable. For example, in the telic sentence “Mamá cierra la puerta/Mom closes the door”, the video shows the same room where the door is present, along with the same furniture. In the coherent version, Mom is seen walking toward the door and closing it, while in the incoherent version, the door remains open, and Mom is sitting in the chair, knitting clothes. Similarly, in the atelic sentence “Mamá pinta la puerta/Mom paints the door,” the coherent version shows Mom in the same living room painting the door with a brush, while in the incoherent version, a closed bucket of paint is present, the door remains unpainted, and Mom is sitting and knitting instead (see Table 2).

It should be noted that for the creation of the linguistic material, the maximum degree of closure of the action was considered [33]. A written survey was conducted with university students studying education. They rated the sentences according to their continuity or completion. Significant differences were found (t = 12.86, *p* < 0.001) between telic sentences (e.g., “Mom turns on the oven”) and atelic sentences (e.g., “Mom cleans the oven”). According to the normative study, the sentences with the highest sense of closure were selected for the telic actions, and those with the highest continuity for the atelic ones. This distinction was reinforced by the accompanying videos, in which the action depicted in the coherent telic sentences had a clear end, whereas in the atelic sentences the action was still being depicted.

The subjects of the sentences included four family members: Mom, Dad, Emma, and Charlie. Direct objects and verbs were controlled for frequency (t = 0.359, *p* = 0.723) and length (t = 0.372, *p* = 0.713). The sentences were recorded to ensure that the duration of each segment was consistent across all stimuli.

The sentences were recorded in sections to control the duration of each segment. Microsoft Clipchamp [34] was used for this purpose. The accompanying videos had an average length of 4 s and were created using the stop-motion technique with images that did not differ in color or size. The images were made in PowerPoint [35] and then recorded into a video in the same application. Expert judgment was used to assess the coherence of the videos, ensuring they accurately reflected the two variables (coherent, incoherent) and that the action depicted was clearly ongoing in the present, aligning with the use of present-tense verbs in the sentences. Examples of each condition are presented in Table 2.

#### 2.2.2. Task

The task consisted of viewing a video accompanied by an oral and written sentence that may or may not agree with the video.

**Figure 1:**
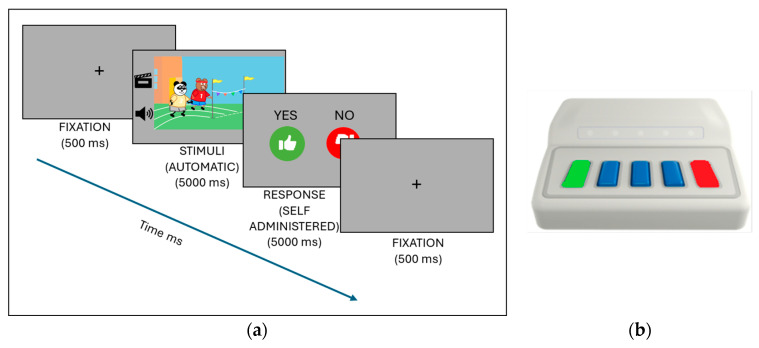
(**a**) Presentation stimuli. (**b**) Chronos device.

Participants received instructions through an interactive drawing that introduced them to the family and the task: “This is the Bear family: Dad, Mom, Emma and Charlie. They have a problem; they can’t remember if they did an action or not. To help them, you have to press the yes button (green) if you think they did the action and the no button (red) if they didn’t”. After receiving the instructions, participants completed four practice stimuli before beginning the experimental phase (Figure 1a). The experimental phase was divided into two blocks, each containing 16 telic–coherent, 16 telic–incoherent, 16 atelic–coherent, and 16 atelic–incoherent stimuli presented in a random order.

The stimuli were presented electronically using E-Prime 3.0 software [36], and for the response, the USB response device Chronos [37] was used. This was adapted so that the two outside buttons could be used for yes and no (Figure 1b).

#### 2.2.3. EEG Recording

EEG data were recorded continuously at a sampling rate of 500 Hz using 32 cap-mounted active electrodes (actiCAP) from Brain Products GmbH (Gilching, Germany), positioned according to the international 10/20 system. The electrodes covered the frontal, parietal, temporal, and occipital lobes, specifically at the following sites: FP1, FP2, F3, F4, F7, F8, Fz, FC1, FC2, FC5, FC6, FT9, FT10, C3, C4, Cz, CP1, CP2, CP5, CP6, P3, P4, Pz, T7, T8, TP9, TP10, P7, P8, O1, O2, and Oz. FCz was used as a reference electrode and the ground electrode was located on the forehead.

Before each recording, preparation included impedance measurements to ensure that all electrode impedances were below 25 KΩ. The Brain Products acquisition system consists of a battery-powered amplifier and fiber-optic communication to the acquisition PC to minimize external electrical noise contamination.

**Figure 2:**
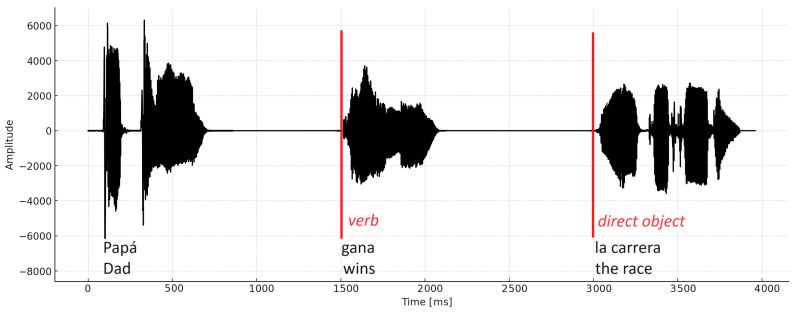
Waveform of the audio file (mono) corresponding to the sentence. The red lines indicate the beginning of the areas of analysis: verb and direct object.

Using BrainVision Analyzer software version 2.3, the recorded data were pre-processed according to standard practices. Data were filtered using an 8th-order Butterworth bandpass filter, with cut-off frequencies of 0.1 Hz and 30 Hz. Artifacts were rejected by visual inspection from an expert. Both verb and direct object ERP were obtained. Stimulus phrases were designed so that the verb segment and the direct object segment started at specific times, as shown in the example in Figure 2.

The verb and direct object segments were standardized to 1200 ms (−200 ms to 1000 ms). The verb segment was obtained from 1300 to 2500 ms and the direct object from 2800 to 4000 ms from the EEG recorded and synchronized to the start of the auditory and visual stimuli.

#### 2.2.4. Analysis

Before the analysis, a visual inspection using a semiautomatic method was conducted to detect and reject artifacts. The thresholds used for the inspection were 150 µV maximum deviation and a gradient of 50 µV/ms. After visual inspection, ICA correction using FP2 (vertical) and F8 (horizontal) references for eye-movement artifacts was applied.

ERP analysis was conducted in R version 4.3 [38]. The segments were baseline-corrected in the interval between −200 and 0 ms. First, a point-by-point Student’s *t*-test was conducted for each segment to detect intervals with significant differences between groups, telicity, and coherence. The selected segments to be studied were 250–500 ms and 500–700 ms in the verb segment, and 250–500 ms and 600–1000 ms in the direct object segment.

For the 4 segments selected, and for each EEG signal channel, a general ANOVA between group (DLD and TD), telicity (telic or atelic verb), coherence (coherent or incoherent), hemisphere (left, right, and center), and region (frontal, central, parietal, and temporal) was conducted, comparing segment means. The significance level was set at 5%. In the cases where significant differences were detected, an ROI exploration was conducted by combining neighboring electrodes.

## 3. Results

### 3.1. ERP Results

#### 3.1.1. N400 Effects in Verb Segment

**Figure 3:**
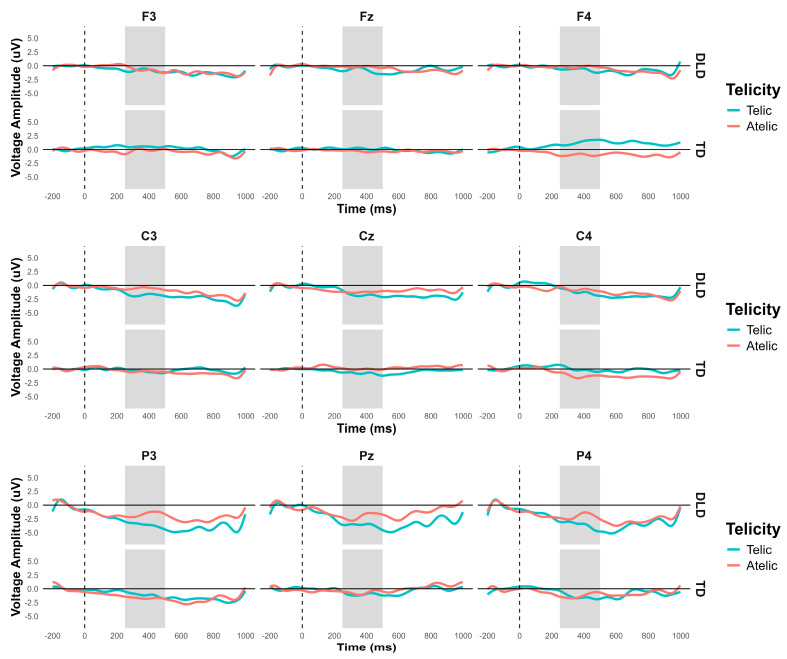
N400 grand average ERP for channels F3, Fz, F4, C3, Cz, C4, P3, Pz, and P4, representing the ANOVA effects in verb segment. Telic conditions are in light-blue color and atelic conditions are in red.

**Figure 4:**
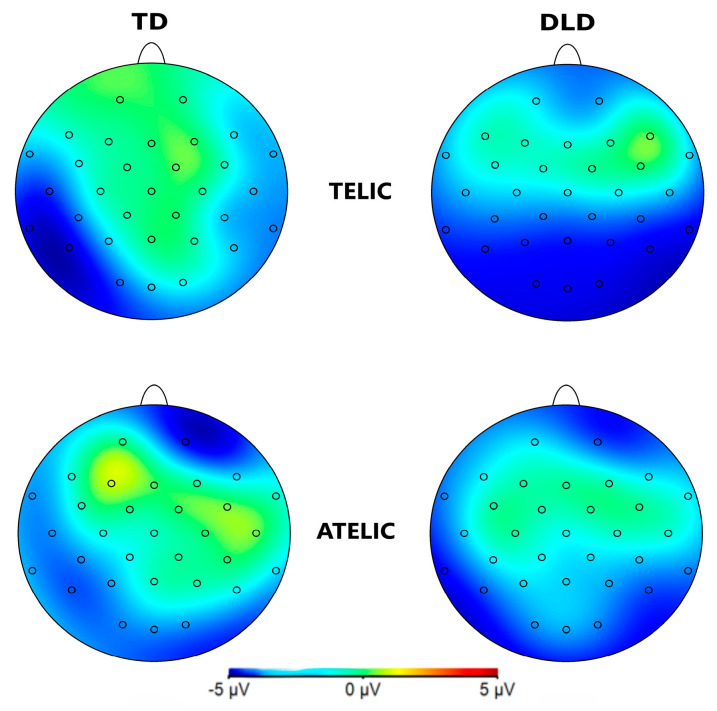
Topographical map of N400 component for verbs in the 250–500 ms time window for the telic and atelic (telicity) conditions between groups (TD and DLD).

The general ANOVA in the time window of 250 to 500 ms revealed a significant interaction between group and telicity—F (1354) = 4.705, *p* = 0.030—where children with DLD showed a more negative amplitude in the telic condition compared to TD children (see Figure 3 and Figure 4). Specifically, comparisons between the DLD and TD groups showed significant differences in both the telic and atelic conditions. For telic conditions, the difference between the TD and DLD groups was M = 1.307, SE = 0.19, t (3596) = 6.881, *p* < 0.001. For atelic conditions, the difference was M = 0.733, SE = 0.19, t (3596) = 3.859, *p* < 0.001. Within the DLD group, there was a significant contrast between atelic and telic conditions, where the telic condition showed a more negative amplitude than the atelic, with a contrast estimate of M = 0.712, SE = 0.19, t (3596) = 3.748, *p* < 0.001. No significant contrast was found within the TD group: M = 0.712, SE = 0.19, t (3596) = 0.727, *p* = 0.467.

Additionally, there was a significant interaction between group and coherence: F (1354) = 6.523, *p* = 0.011. However, we did not explore this effect further since, at this point in the stimulus, children were not able to detect the coherence or incoherence yet.

#### 3.1.2. Post-N400 Effects in Verb Segment

**Figure 5:**
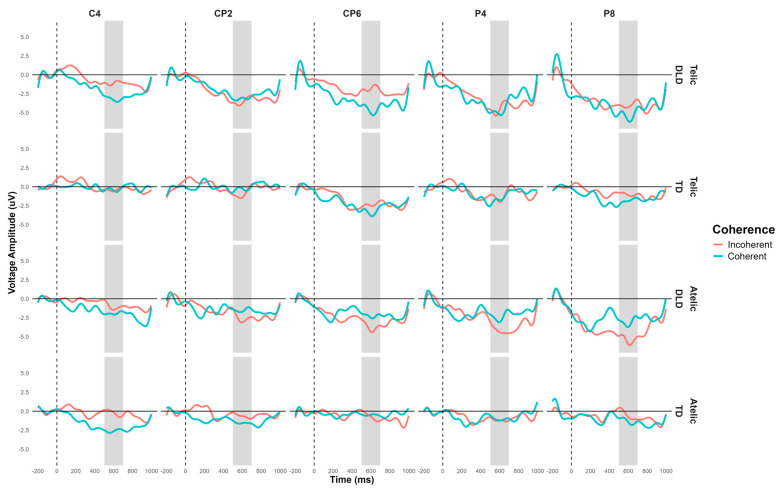
Post-N400 grand-average ERP for channels C4, CP2, CP6, P4, and P8 forming the right centroparietal ROI in verb segment. Coherent conditions are in light-blue color and incoherent conditions are in red.

**Figure 6:**
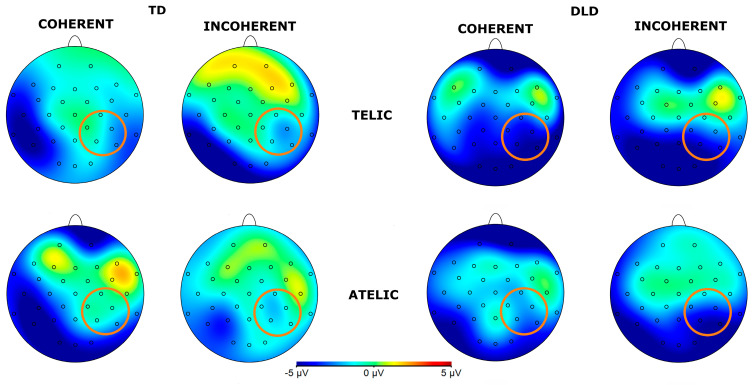
Topographical map of post-N400 component for verbs in the 500–700 ms time window for telicity (telic and atelic) and coherence (coherent and incoherent) conditions between groups (TD and DLD) for ROI involving channels C4, CP2, CP6, P4, and P8 (see orange circles for ROI).

The general ANOVA of the time window of 500 to 700 indicated a significant triple interaction between group, telicity, and coherence: F (1354) = 4.347, *p* = 0.0371. We focus on the right centroparietal ROI composed of electrodes C4, CP2, CP6, P4, and P8: F (1, 712) = 4.328, *p* = 0.038. This region was selected based on theoretical considerations, as previous research has indicated that the centroparietal areas are critically involved in the processing of semantic and syntactic information [39]. The intergroup contrasts in this region revealed significant differences in several conditions. For telic–coherent sentences, children with DLD showed a more negative amplitude than the TD group; the mean difference was M = 2.746, SE = 0.711, t (712) = 3.865, *p* < 0.001. In telic–incoherent sentences, although both groups processed incoherence more positively, this effect showed greater amplitude in the DLD group; the difference was M = 1.726, SE = 0.711, t (712) = 2.429, *p* = 0.015. For atelic–incoherent sentences, children with DLD showed a more negative amplitude than the TD group: M = 2.710, SE = 0.711, t (712) = 3.813, *p* < 0.001 (see Figure 5 and Figure 6). However, the atelic–coherent condition was not significant between the groups (M = 0.773, SE = 0.711, t (712) = 1.088, *p* = 0.277). In contrast, the intragroup comparisons did not reveal significant differences. The ERP waveforms show distinct patterns of neural activity between the groups across these conditions, highlighting the interaction effects observed.

#### 3.1.3. N400 Effects in Direct Object Segment

**Figure 7:**
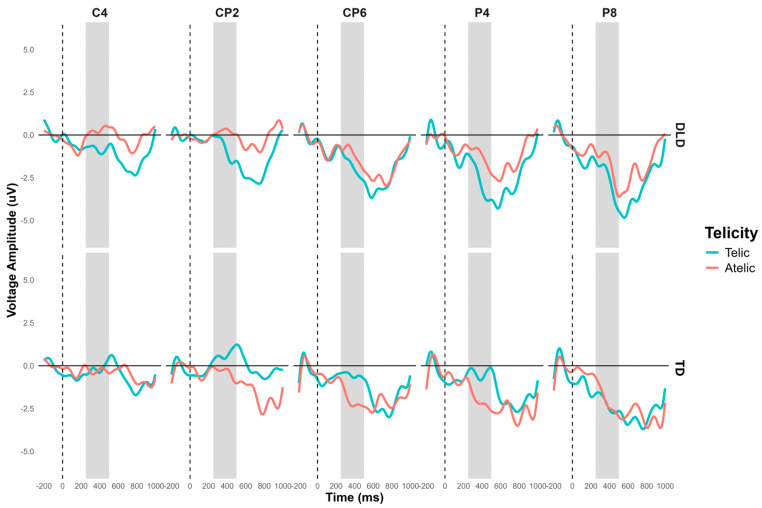
N400 grand average ERP for channels C4, CP2, CP6, P4, and P8 forming the right centroparietal ROI for object segment. Telic conditions are in light-blue color and atelic conditions are in red.

The general ANOVA in the time window of 250 to 500 ms showed a significant interaction between group and telicity: F (13,728) = 13.504, *p* < 0.001. As mentioned previously, we focus on the right centroparietal ROI composed of electrodes C4, CP2, CP6, P4, and P8: F (1, 752) = 9.702, *p* < 0.001. The contrast in this region shows that, in the telic condition, children with DLD showed a more negative amplitude than TD children: M = −1.184, SE = 0.409, t (756) = −2.899, *p* < 0.001. In the atelic condition, the difference was not significant: M = 0.614, SE = 0.409, t (756) = 1.503, *p* = 0.133. Intragroup contrasts for the DLD group showed a more negative amplitude in the telic condition than the atelic condition: M = 1.010, SE = 0.409, t (756) = 2.472, *p* = 0.014. For the TD group, the difference between telic and atelic conditions was marginally significant, showing a more negative trend in the atelic condition compared to the telic condition: M = −0.789, SE = 0.409, t (756) = −1.931, *p* = 0.054 (see Figure 7). The ERP waveforms highlight the significant differences observed, particularly in the telic condition between groups.

**Figure 8:**
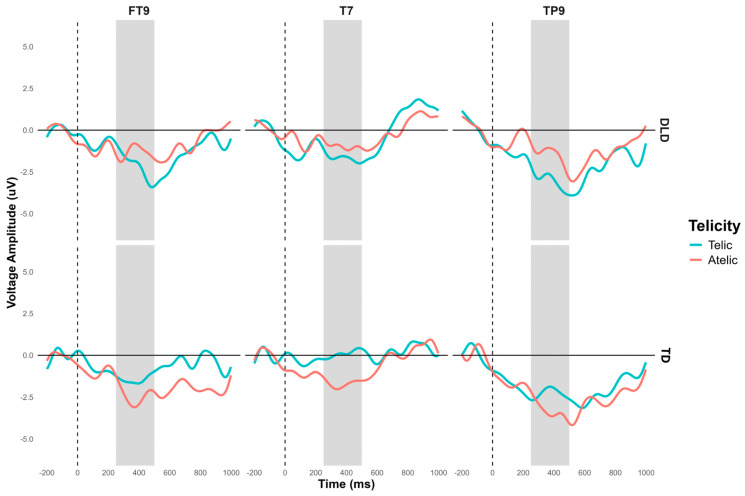
N400 grand average ERP for channels FT9, T7, and TP9 forming the left temporal ROI for object segment. Telic conditions are in light-blue color and atelic conditions are in red.

**Figure 9:**
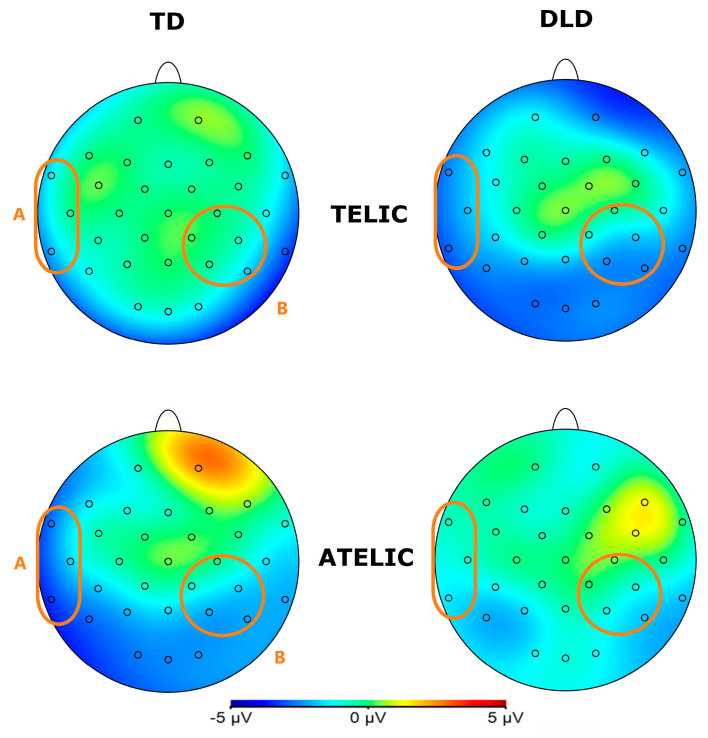
Topographical map of N400 component for object segment in the 250–500 ms time window for the telic and atelic (telicity) conditions between groups (TD and DLD). A: ROI involving channels FT9, T7, and TP10; B: ROI involving channels C4, CP2, CP6, P4, and P8.

Additionally, a significant interaction between group and telicity was observed in the left temporal region composed of electrodes FT9, T7, and TP9: F (1, 448) = 5.804, *p* = 0.016. The contrast in this region approaches significance in several conditions. In the telic condition, both groups show, in general, a negativity that is slightly more pronounced in children with DLD: M = −1.09, SE = 0.677, t (452) = −1.604, *p* = 0.109. In the atelic condition, the difference is marginally significant: M = 1.23, SE = 0.677, t (452) = 1.814, *p* = 0.07. Intragroup contrasts for the TD group show a marginally significant difference between the telic and atelic conditions: M = −1.30, SE = 0.677, t (452) = −1.913, *p* = 0.056. This interaction shows that TD children showed different neural responses in this region compared to the DLD group (see Figure 8 and Figure 9).

#### 3.1.4. P600 Effects in Direct Object Segment

**Figure 10:**
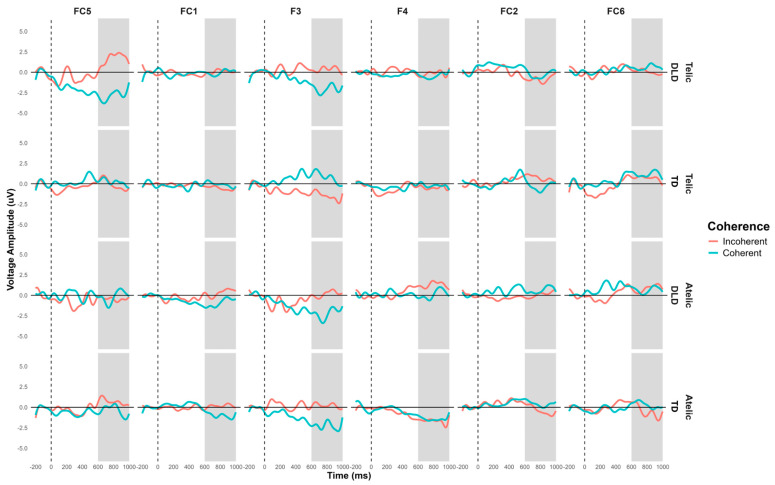
Grand average ERP for channels F3, FC1, FC5, F4, FC2, and FC6 forming the frontal ROI for object segment. Coherent conditions are in light-blue color and incoherent conditions are in red.

**Figure 11:**
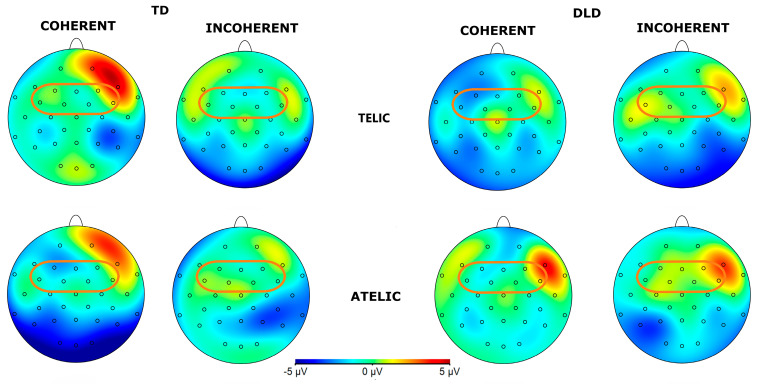
Topographical map of P600 component for object segment in the 600–1000 ms time window for telicity (telic and atelic) and coherence (coherent and incoherent) conditions between groups (TD and DLD) in ROI involving channels F3, FC1, FC5, F4, FC2, and FC6 (see orange circles for ROI).

The general ANOVA in the time window of 600 to 1000 ms revealed a significant interaction between group, telicity, coherence, and hemisphere: F (13,728) = 6.525, *p* = 0.0107. When we focus on the frontal ROI conformed by F3, FC1, FC5, F4, FC2, and FC6 (F (1, 896) = 5.127, *p* = 0.024) the intergroup contrasts indicate significant differences in several conditions. For telic–coherent sentences in the left hemisphere, children with DLD show a more negative amplitude than TD children: M = −1.969, SE = 0.814, t (896) = −2.419, *p* = 0.016. In telic–incoherent sentences in the left hemisphere, the difference is marginally significant: M = 1.455, SE = 0.814, t (896) = 1.787, *p* = 0.074. For atelic–incoherent sentences in the right hemisphere, the difference is also significant (M = 1.631, SE = 0.814, t (896) = 2.004, *p* = 0.045), showing a more negative trend in the TD group. The intragroup contrasts for the DLD group showed significant differences between the telic–coherent and telic–incoherent conditions in the left hemisphere (M = −2.3834, SE = 0.814, t (896) = −2.928, *p* = 0.018), showing a more negative amplitude in the telic–coherent condition. No significant differences were found in the TD group. The ERP waveforms illustrate these differences, particularly highlighting the interaction effects observed across hemispheres and conditions (see Figure 10 and Figure 11). Similar effects were found with a more frontal distribution in studies involving similar semantic tasks, focusing specifically on object–verb manipulation in the Spanish language [40].

### 3.2. Behavioral Results

The behavioral analyses were performed with R [38]. To ensure data accuracy, outliers were removed using a criterion of two standard deviations from the mean. This approach effectively eliminated data points significantly deviating from the norm [41]. Approximately 8% of the reaction time (RT) data points in the entire sample were identified as outliers.

**Figure 12:**
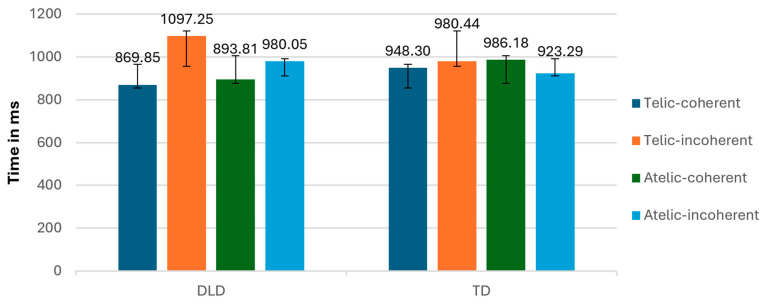
Mean reaction times (RTs; ±SE) according to conditions and group.

The results of the repeated-measures ANOVA revealed significant interactions. A significant interaction was observed between group and coherence (F (1, 34) = 7.130, *p* < 0.001), suggesting that the difference in response to coherent and incoherent verbs varied between the DLD and TD groups (Figure 12). The DLD group showed longer reaction times when the condition was incoherent and shorter reaction times when the condition was coherent, in comparison to the TD group. The following analysis of independent sample t-tests indicated no significant interaction.

At the intragroup level, the DLD group showed longer reaction times for incoherent conditions compared to coherent conditions, indicating greater difficulty or slower processing when the information presented was not coherent. In contrast, the TD group exhibited faster reaction times overall, with less pronounced differences between coherent and incoherent conditions, suggesting more efficient processing and better ability to handle both coherent and incoherent information.

Additionally, the interaction between telicity and coherence was significant (F (1, 34) = 7.348, *p* = 0.012), indicating that the combination of telicity and coherence affected participants differently.

**Figure 13:**
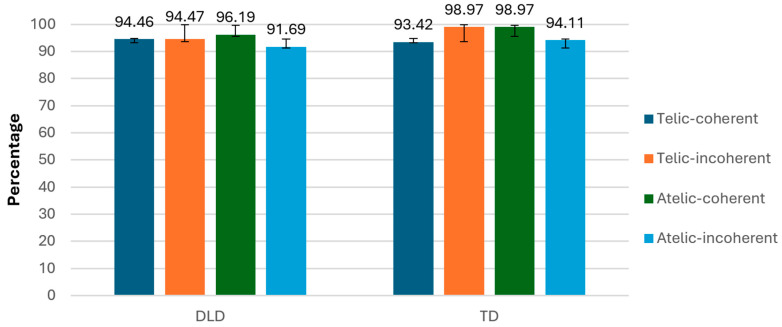
Accuracy (%; ±SE) according to conditions and group.

In terms of accuracy, we found a significant interaction between telicity and coherence (F (1, 34) = 14.576, *p* < 0.001), indicating that the combination of telicity and coherence affected accuracy differently to the conditions (Figure 13).

## 4. Discussion

The current study investigated the differences in the neural and linguistic processing of telic and atelic verbs in Spanish-speaking children with DLD compared to their TD peers. An original experimental design was used, featuring sentences and images that expressed actions coherent with an endpoint or an ambiguous point of development, contrasted with actions incoherent with the telic and atelic semantics.

According to the proposed hypothesis, we expected to find difficulties in children with DLD in distinguishing atelic verbs in contrast to telic verbs through an attenuation of the N400 for incoherent atelic sentences. These assumptions were based on findings in English- and German-speaking speaking populations, where atelic verbs are processed later and with greater difficulty than telic verbs [20,23].

### 4.1. Discussion of Verb Results

Based on the Event Structural Bootstrapping model, children with DLD deviate from normal language acquisition routes, seeking compensatory strategies such as overgeneralization in the semantic representation of verbs, which would lead to difficulties in distinguishing telic and atelic verbs [22,26]. These assumptions are confirmed by the results found in relation to the verbs of the experimental sentences through the N400 component. The significant double interaction of telicity by group in the verb segment establishes that TD children show greater difficulty in integrating the linguistic meaning of atelic verbs, indicated by a greater amplitude of the N400 component, while the DLD group shows the opposite effect, with greater difficulty in processing telic verbs compared to atelic verbs. This inverse effect might be due to the telicity in the present verb generating an effect of an unfinished action that could be affecting children with DLD [26].

The contrast analysis indicated significant differences between telic and atelic verbs in both groups, clearly showing opposing intergroup trends. At the intragroup level, only the DLD group showed significant differences between telic and atelic verbs, with a higher cognitive cost for telic verbs. According to the literature, the past tense fits better and seems easier with telic verbs that imply a completed action than with the present tense, as used in the current experiment [25,26,42]. When the present tense is used with telic verbs, their degree of telicity decreases, creating an imperfect aspect of the action, while progressive and present-tense contexts may be easier with atelic verbs because the action is ongoing. These temporal differences could generate a semantic effect in children with DLD with difficulties in processing tense markers and the telicity of a verb [42].

The telicity effect manifested early in both groups; however, the incoherent atelicity that the TD group detected from 250 ms, the DLD group detected in the later window of 500–700 ms. This effect could be interpreted as a post-N400 component, aligning with the hypothesis that the processing time for telicity in children with DLD starts later [29]. This delay in processing is consistent with recent eye-tracking studies on Spanish verbal tense comprehension, which suggest that while children with DLD may understand tense morphology, they experience delays in processing that could contribute to a slower integration of tense information in real time [27]. On the other hand, in terms of telicity, the TD group detected the difficulty of present-tense verbs afterwards as a more semantic processing of the verb, although no significant intragroup differences were observed in the experimental variables. It is noteworthy that the telic and atelic effects in children with DLD were generally much more negative than in TD children, indicating the cognitive cost of telicity for this population.

Similar results were found on the lexical–semantic condition in the study by Courteau et al. [43], who identified the typical N400 component associated with increased lexico-semantic processing difficulty at 500–700 ms (post-N400), indicating additional post-lexical integration processes. In the same vein, in a recent investigation, Courteau et al. [30] reported that lexico-semantic mismatches elicited broadly distributed N400-like negativities via centro-parietal electrodes in TD and DLD groups, with the N400 duration extending beyond the classical 300–500 ms window typical for reading studies. However, both studies were conducted in adults and adolescents, respectively; therefore, there could be differences in lexical–semantic processing in children.

Kornilov et al. [44] investigated lexical processing deficits in 23 Russian-speaking children with DLD compared to 16 TD peers using a picture–word matching paradigm. According to their results, lexico-semantic mismatches elicited significantly attenuated N400 amplitudes in children with DLD compared to TD children in the semantically unrelated and initial phonological overlap conditions, indicating deficits in processing both phonological and lexico-semantic mismatches. The N400 component had a prominent parietal distribution, particularly over the midline parietal (Pz, PO3, PO4, POz, Oz) and right parietal (P4, P6, PO8) electrode clusters. On the other hand, in the study by Pijnacker et al. [29] on preschoolers with DLD in comparison with TD peers, where the task involved listening to sentences containing either congruent or incongruent words, the N400 component, typically associated with semantic processing, was analyzed in two time windows: 300–500 ms and 500–800 ms. The TD group exhibited a robust N400 effect in both time windows, particularly over posterior electrode sites (e.g., Pz, P3, P4, CPz). In contrast, the DLD group only showed a significant N400 effect in the later 500–800 ms window, indicating delayed semantic processing capability. These results are directly related to the findings found in the present study. However, both studies were conducted in languages other than Spanish, hence the relevance of this study in the field.

### 4.2. Discussion of Direct Object Results

In the case of the direct object segment, in the early window of 250 to 500 ms, our analysis revealed a significant interaction between group and telicity. Specifically, the DLD group showed significantly different neural responses compared to the TD group when processing telic direct objects, with a higher cognitive cost associated with telic verbs. The observed N400 effect in the verb window continued to be present at this level, suggesting a persistent difficulty with telicity due to the present-tense conjugation, which may affect the recognition of this component. In the TD group, atelic verbs began to show a more negative response towards the end of the sentence, with the effect localized in the right parietal region, as expected from the previous literature [43]. This interaction highlights the distinct neural mechanisms between the groups, where children with DLD show a less efficient processing of telic linguistic elements.

Additionally, the significant interaction found in the left temporal region can likely be attributed to the multimodal nature of the task, as the sentences were listened to by the participants. Significant interactions in this area are expected due to the auditory processing involved [45]. This activation suggests that children with DLD may face challenges not only in semantic integration, but also in the auditory processing of the sentences, potentially complicating their ability to handle telic verbs in the present tense. This is consistent with the findings of Malins et al. [46], who investigated the neural mechanisms underlying auditory word recognition in children with DLD compared to TD children. Their study highlighted differences in the N400 response, indicating that children with DLD exhibit atypical lexical processing. Furthermore, Malins et al. [46] emphasized the importance of the temporal region in early auditory processing and its role in subsequent phonological and lexical processing, noting that these differences can influence later stages of word recognition.

In the subsequent window of 600 to 1000 ms, our analysis revealed a significant interaction between group, telicity, coherence, and hemisphere. For telic–coherent and –incoherent sentences, children with DLD detect the effect of telicity later than their TD peers. These results are consistent with the hypothesis that children with DLD will show a delayed effect in response to incoherent sentences [29]. In this case, the effects were detected in a later time window. On the other hand, the TD group seems to engage in a review of the coherence of atelic sentences, resulting in increased negativity. This suggests that, at this stage, TD children are re-evaluating whether the sentence accurately describes an event that occurred, contributing to a higher cognitive cost. Children with DLD, however, do not appear to be performing this re-evaluation, indicating potential difficulties in their ability to reassess sentence coherence.

As discussed in the introduction, children with DLD appear to exhibit asymmetry at the developmental level, which could be reflected in these findings [9]. Functional MRI studies reveal atypical brain activation patterns in children with DLD, including hypoactivation in the left inferior frontal gyrus (IFG) and compensatory activation in the right IFG [9].

This could explain why children with DLD elicited more negativity in the telic–coherent condition compared to TD children in the left hemisphere, whereas the same population showed more negativity in the atelic–incoherent condition compared to the TD population. This right hemisphere activation in the DLD group may indicate compensatory neural mechanisms due to their atypical neural development.

In sum, the delayed detection of telicity with a greater cognitive burden in coherent contexts i.e., at the end of sentences and the right hemisphere engagement in more complex experimental conditions explain the difficulties in distinguishing between telic and atelic verbs, a challenge that may be related to overgeneralization strategies in the population of children with DLD [25,26]. These findings underscore the unique challenges faced by children with DLD in processing complex linguistic information.

### 4.3. Discussion of Behavioral Results

The behavioral task in this study involved a semantic judgment related to the action depicted in the sentences. In analyzing the behavioral results, it is important to consider that while reaction times (RT) offer mediated access to underlying mental processes, ERP is a true online method that provides immediate access to these processes [28]. This difference might mean that the effects of telicity were not as pronounced in the RT data as they were in the ERP data. This is reflected in the significant interaction between group and coherence in reaction time, which is the only significant interaction observed. The nature of the RT task could potentially contribute to the attenuation of the telicity effect in the reaction times.

Children with DLD showed a trend of more heterogeneous reaction times, suggesting a potential greater difficulty in recognizing the telicity of verbs compared to their TD peers. This variability in RT may indicate that children with DLD struggle more with processing the endpoint of telic actions, which aligns with the ERP results showing a delayed detection of telicity. However, these behavioral results are difficult to interpret with confidence, as they may not fully capture the nuanced processing difficulties that are more clearly revealed through the EEG technique.

Interestingly, the DLD group also exhibited faster reaction times in some conditions, which could be attributed to impulsivity. This faster response time might not necessarily indicate better processing, but rather a tendency to respond quickly without thorough processing of the semantic content. Zapparrata et al. [47], in their meta-analysis of time-based tasks in children with DLD, found that individuals with DLD often exhibit slower processing across various tasks. However, their analysis also highlighted that, in some contexts, this slow processing can be masked by impulsive responses. Children with DLD might prioritize speed over accuracy, leading to faster reaction times that do not reflect efficient or accurate semantic processing [47].

## 5. Conclusions

This study investigated the neural and linguistic processing of telic and atelic verbs in Spanish-speaking children with Developmental Language Disorder (DLD) compared to their typically developing (TD) peers. Using ERP measures and behavioral tasks, we identified distinct patterns of verb processing in these groups.

Our findings highlight that children with DLD exhibit greater difficulty in processing telic verbs, likely due to challenges in integrating semantic and temporal information. This was evident in the N400 component, where children with DLD showed a delayed and attenuated response compared to TD children.

Overall, this research provides valuable insights into the neural and behavioral correlates of verb processing in children with DLD, emphasizing the importance of these findings for understanding the acquisition and development of language in this population. By the age of 7, children with DLD are expected to have an understanding of telicity that typically develops around the age of 4 [21]. This underscores the need for early and targeted interventions that focus on the semantics of verbs at an early stage to support language development in children with DLD. Current interventions by specialists often focus more on general aspects of semantics rather than specifically targeting the development of telicity [48]. Promoting the development of telicity in children with DLD may be critical to their overall language acquisition and proficiency.

## Figures and Tables

**Table 1:** Participant characteristics.

Variable	DLD (*n* = 18)			TD (*n* = 18)			Comparison
	M	SD	Min–Max	M	SD	Min–Max	
Age (months)	91.83	3.70	83–95	91.78	4.50	83–97	t = −0.04 *p* = 0.98
IDTEL Score	56.39	6.71	38–66	104.56	13.70	83–124	t = −13.39 *p* = <0.001

Note: Group mean values (M), standard deviation (SD), and score from IDTEL.

**Table 2:** Item examples.

Variable	Telic–Coherent	Telic–Incoherent	Atelic–Coherent	Atelic–Incoherent
Sentence	Papá gana la carrera/Dad wins the race	Papá gana la carrera/Dad wins the race	Papá corre la carrera/Dad runs the race	Papá corre la carrera/Dad runs the race
Video	Dad crosses the finish line	Another runner wins, dad is right behind him	Dad comes running	Dad is sitting watching the race
Sentence	Mamá enchufa el secador/Mom plugs in the hairdryer	Mamá enchufa el secador/Mom plugs in the hairdryer	Mamá usa el secador/Mom uses the hairdryer	Mamá usa el secador/Mom uses the hairdryer
Video	She picks up the cord and plugs it in	She applies perfume, and the hairdryer is on the vanity	She applies perfume, and the hairdryer is on the vanity	She applies perfume, and the hairdryer is on the vanity
Sentence	Emma rompe el celular/Emma breaks the phone	Emma rompe el celular/Emma breaks the phone	Emma revisa el celular/Emma checks the phone	Emma revisa el celular/Emma checks the phone
Video	She throws the phone on the ground and it breaks	She puts the phone in her bag without looking	She has the phone in her hands and checks it	She reads a book without looking at the phone
Sentence	Charlie mancha el sillón/Charlie stains the couch	Charlie mancha el sillón/Charlie stains the couch	Charlie duerme en el sillón/Charlie sleeps on the couch	Charlie duerme en el sillón/Charlie sleeps on the couch
Video	He spills ice cream on the couch	He plays with blocks on the couch	He sleeps on the couch	He jumps on the couch

## Data Availability

The data generated and analyzed in this study are available on reasonable request from the corresponding author. These data are not publicly available as they are human data from adults and children in neurotypical and clinical groups.
